# Implementation challenges and lessons learned from the STREAM clinical trial—a survey of trial sites

**DOI:** 10.1186/s13063-023-07068-8

**Published:** 2023-01-23

**Authors:** Leena N. Patel, Meera Gurumurthy, Gay Bronson, Karen Sanders, I. D. Rusen

**Affiliations:** 1 Vital Strategies, 100 Broadway, 4th Floor, New York, NY 10005 USA; 2 Vital Strategies Health Systems, Asia Pacific, Singapore, Singapore; 3 Medical Research Council Clinical Trials Unit at UCL, London, England

**Keywords:** MDR-TB, Trial implementation, Challenges, Lessons learned, Best practices, Site perspective

## Abstract

**Background:**

Design and implementation of multi-country clinical trials for multidrug-resistant tuberculosis (MDR-TB) are complex for several reasons, including trial duration, varying levels of experience and infrastructure across settings, and different regulatory requirements. STREAM was an MDR-TB clinical trial that recruited over 1000 participants. We documented challenges and best practices/lessons learned from the site perspective to improve implementation of future trials.

**Methods:**

We conducted a voluntary survey of trial staff at all sites to obtain information on challenges encountered and best practices/lessons learned from implementation of the STREAM trial. Respondents were asked to identify substantive aspects of trial implementation from a list that included: trial administration, laboratory strengthening/infrastructure, pharmacy and supply chain management, community engagement, regulatory and ethics requirements, health economics, and other (respondent designated) about which a practical guide would be useful to improve future trial implementation. For each aspect of trial implementation selected, respondents were asked to report challenges and best practices/lessons learned during STREAM. Lastly, respondents were asked to list up to three things they would do differently when implementing future trials. Summary statistics were generated for quantitative data and thematic analysis was undertaken for qualitative data.

**Results:**

Of 67 responses received from 13 of 15 sites, 47 (70%) were included in the analyses, after excluding duplicate or incomplete responses. Approximately half the respondents were investigators or trial coordinators. The top three aspects of trial implementation identified for a best practices/lessons learned practical guide to improve future trial implementation were: trial administration, community engagement, and laboratory strengthening/infrastructure. For both challenges and best practices/lessons learned, three common themes were identified across different aspects of trial implementation. Investment in capacity building and ongoing monitoring; investment in infrastructure and well-designed trial processes; and communication and coordination between staff and meaningful engagement of stakeholders were all thought to be critical to successful trial implementation.

**Conclusions:**

Existing practices for clinical trial implementation should be reevaluated. Sponsors should consider the local context and the need to increase upfront investment in the cross-cutting thematic areas identified to improve trial implementation.

**Supplementary Information:**

The online version contains supplementary material available at 10.1186/s13063-023-07068-8.

## Introduction

Design and implementation of multidrug-resistant tuberculosis (MDR-TB) clinical trials are complex. Several factors need to be considered when designing an MDR-TB clinical trial from selection of a control regimen and outcome measure(s) for efficacy, to duration and frequency of follow-up [1]. The duration of MDR-TB clinical trials poses challenges in terms of resource requirements, given the length of treatment regimens and the follow-up period required to evaluate for relapse. Identifying sufficient eligible participants at any single center is also difficult, often requiring multi-center studies [2]. Importantly, global registration of new regimens requires clinical trial evaluations in appropriately diverse populations [3]. Conducting multi-center studies across settings adds to the complexity due to different regulatory requirements and approval processes [4]. In low-resource settings, barriers to conducting clinical trials include constraints in financial and human capacity, ethical and regulatory system obstacles, lack of a research environment, operational barriers, and competing demands [5]. Although the tuberculosis (TB) drug pipeline has improved over the past decade, the lack of new drug development for over 40 years prior to that means that few sites and laboratories have significant experience conducting clinical trials for registration and in accordance with internationally accepted standards of good clinical practice and good laboratory practice [3].

Globally, less than one-third of randomized clinical trials registered from 2010 to 2019 were set in low- and middle-income countries (LMICs), including only 5% in south Asia and 2% in sub-Saharan Africa [6]. These regions have some of the highest burdens of TB disease [7], but research infrastructure and capacity to conduct clinical trials is variable across settings and often needs to be strengthened [8], especially in LMICs that have the highest burden of TB disease [3]. The limited clinical research experience in these regions, specifically in the field of MDR-TB, can result in delays in approvals and raises other logistical obstacles to the successful initiation, and ongoing implementation of clinical trials [9]. An assessment conducted by the TB Alliance in 39 countries found that 51 (62.2%) of trial sites and their associated mycobacteriology laboratories had the potential to be ready for a registration trial within 12 months suggesting that significant capacity building was required with concerted, appropriately-resourced efforts [10].

Early engagement with experienced sites, establishing relationships with key stakeholders, providing adequate resources and support for inexperienced sites, and partnering with contract research organizations (CROs) can impact the success of trials [9], but a systematic review found that although health research capacity in LMICs has improved, barriers persist and more evidence on capacity development strategies is needed [11]. In addition, while challenges from the sponsor or CRO perspective have been documented, there is limited literature from the perspective of participating sites to inform future decisions around trial implementation.

The Evaluation of a Standardized Treatment Regimen of Anti-tuberculosis Drugs for Patients with Multidrug-resistant Tuberculosis (STREAM) is a multi-country clinical trial evaluating shortened regimens for MDR-TB. STREAM enrolled more than 1000 participants over an 8-year period across two stages, making it the world’s largest recruited clinical trial for MDR-TB. It is also the first phase III trial to test the efficacy and safety of bedaquiline within a shortened treatment regimen.

Stage 1 of the trial, implemented at seven sites in Ethiopia, Mongolia, South Africa, and Vietnam, was completed in 2017 and primary outcome results at 132 weeks of follow-up were published in 2019 [12]. Stage 2 of the trial commenced in 2016 and was implemented at 13 sites in Ethiopia, India, Georgia, Moldova, Mongolia, South Africa, and Uganda with primary outcome results at 76 weeks of follow-up published in November 2022 [13].

In total, 15 unique sites participated across the two stages of STREAM. All sites had experience treating patients with MDR-TB; however, site-level research capacity was variable—some sites were large research centers with prior experience conducting TB clinical trials, whereas some sites had no TB trials management experience in the 5 years prior to initiating STREAM. This paper describes the STREAM experience of trial sites so that knowledge gained and lessons learned from implementation of STREAM are disseminated and inform implementation of future trials.

## Methods

### Data collection

Vital Strategies, the trial sponsor, developed a survey (Appendix 1) to obtain input from sites on different aspects of clinical trial implementation. The survey was disseminated in November 2019 using Survey Monkey, to key personnel, including principal investigators, sub-investigators, and trial coordinators, at all 15 STREAM Stage 1 and 2 sites in 8 countries (Ethiopia, India, Georgia, Moldova, Mongolia, South Africa, Uganda, Vietnam). Key personnel were asked to further disseminate the survey to other site study team members, including pharmacy and laboratory staff, to maximize responses and obtain information from a broad range of respondents. The initial survey response deadline of 1 month was extended to 2 months to increase the response rate.

Respondents’ names were not collected, but they were asked to describe their role and trial site. Respondents were asked to select aspects of trial implementation from a list that included: trial administration; laboratory strengthening/infrastructure; pharmacy and supply chain management; community engagement; regulatory and ethics requirements; health economics; and other (respondent designated), about which a best practices/lessons learned practical guide would be useful to improve implementation of future trials. For each aspect selected, respondents were asked to specify their site’s challenges and best practices/lessons learned from STREAM. To help us formulate practical recommendations, respondents were also asked to list up to three things they would do differently to improve trial implementation if there were another clinical trial at their site.

Respondents were asked to provide informed consent at the start of the survey. The survey was determined to be exempt human subjects research by the Vital Strategies’ Research Subcommittee.

Quantitative and qualitative data, including responses to open-ended questions, were collected in SurveyMonkey, and data were exported to Microsoft Excel for analysis.

### Quantitative and qualitative data analysis

Summary statistics were generated for quantitative data such as the number of sites, proportions of types of respondents, and proportions that selected different aspects of trial implementation about which a guide would be useful.

Qualitative data from open-ended questions including challenges, best practices/lessons learned, and things that respondents would do differently in future trial implementation, were analyzed.

Qualitative data were extracted into separate working sheets and responses from all participants to a particular question were grouped together. Two researchers familiarized themselves with the responses and completed inductive coding (MG and LP). Codes were then grouped into categories from which themes were generated through an iterative process. For questions on challenges and best practices/lessons learned, each researcher independently analyzed qualitative responses to identify themes within each aspect of trial implementation. Themes that emerged from this exercise were reviewed by both researchers to agree on a final set of themes under each aspect of trial implementation. Both researchers then jointly reviewed within-aspect themes to identify common themes that were relevant across multiple aspects of trial implementation for challenges and best practices/lessons learned. Responses to the question regarding changes for future trials were analyzed using the same method. Disagreements and discrepancies were discussed and resolved between the reviewers at various points during the thematic analysis cycle. A third senior researcher (GB) reviewed and discussed interpretations to help identify themes as well as to ensure consistency in the thematic coding process.

Thematic analysis was performed manually using Microsoft Excel to code, categorize, and identify themes for each aspect of implementation for challenges and best practices/lessons learned, as well as to explore emerging cross-cutting themes.

### Researcher characteristics and reflexivity

The authors are experienced in diverse research skills, including both primary quantitative and qualitative research methods. The team reflected on personal biases or judgments throughout the process of the analyses to allow reflection on emerging themes through different perspectives (LP, MG, GB). The authors were either from the trial sponsor organization, or from a coordinating center that oversaw aspects of trial design and implementation; none were from sites that implemented trials.

## Results

A total of 67 respondents consented to participate in the study. After excluding incomplete and duplicate responses (*n* = 19), and complete duplicates (*n* = 1), 47 responses were included in the analysis. Incomplete responses were excluded if information about site and role was provided, but responses to questions about guidebook topics, best practices/lessons learned, challenges, or changes for future trials were not provided. At least one person from 13 (87%) of 15 sites, representing all eight countries participating in the trial, responded to the survey. Five (38%) of the 13 sites did not have TB trial management experience in the 5 years prior to initiating STREAM. Approximately 55% of respondents were from three sites—two in India and one in South Africa (Table 1) and one of these three sites had TB trials management experience in the 5 years prior to initiating STREAM.

**Table 1:** Number of respondents by trial site

Country	Site	Number of respondents (%)
Ethiopia	Site 1	5 (10.6%)
	Site 2	2 (4.3%)
Georgia	Site 1	1 (2.1%)
India	Site 1	10 (21.3%)
	Site 2	6 (12.8%)
	Site 3	1 (2.1%)
Moldova	Site 1	1 (2.1%)
Mongolia	Site 1	3 (6.4%)
South Africa	Site 1	10 (21.3%)
	Site 2	1 (2.1%)
	Site 3	1 (2.1%)
Uganda	Site 1	5 (10.6%)
Vietnam	Site 1	1 (2.1%)

Over 50% of responses were from principal investigators, sub-investigators, and trial coordinators (Table 2).

**Table 2:** Roles of survey respondents in the STREAM clinical trial

Role	***N*** = 47^a^
Principal investigator	11 (23%)
Trial coordinator (includes two respondents with a dual role as sub-investigator/medical officer/physician)	9 (19%)
Sub-investigator/medical officer/physician (includes two respondents with a dual role as trial coordinator/administrator)	6 (13%)
Nurse (includes one respondent with a dual role as data encoder)	2 (4%)
Pharmacist	3 (6%)
Lab technician	3 (6%)
Data manager/encoder (includes one respondent with a dual role as a nurse)	6 (13%)
Field worker	2 (4%)
Administrative (assistant, officer)	3 (6%)
Other^b^	5 (11%)

^a^Total is 50 because two trial coordinators had dual roles as trial physicians, and one nurse had a dual role as a data encoder

^b^Includes DOTS supervisor (*n* = 1), health economist (*n* = 1), counselor (*n* = 1), community liaison officer (*n* = 1), trial drug manager (*n* = 1)

Table 3 summarizes the aspects of trial implementation for which respondents said a best practices/lessons learned practical guide would be useful to improve future trial implementation. Trial administration and community engagement were identified by 51% and 49% of respondents, respectively.

**Table 3:** Aspects of trial implementation about which a best practices/lessons learned guide would be useful

Aspect of trial implementation	N = 47^a^
Trial administration	24 (51%)
Community engagement	23 (49%)
Laboratory strengthening/infrastructure	17 (36%)
Pharmacy and supply chain management	14 (30%)
Regulatory requirements and ethics committee approvals	13 (28%)
Health economics	11 (23%)
Other	10 (21%)

^a^The sum of responses is greater than 47 because respondents could select more than one aspect of trial implementation

### Challenges and best practices/lessons learned

Three cross-cutting challenges and best practices/lessons learned emerged across the different aspects of trial implementation as indicated below. Table 4 captures responses that fit within these three cross-cutting themes.

**Table 4:** Responses categorized into cross-cutting themes for challenges and best practices/lessons learned, by aspect of trial implementation^a^

	Trial administration	Community engagement	Laboratory strengthening/infrastructure	Pharmacy and supply chain management	Regulatory requirements and ethics committee approvals	Health economics	Other^b^
**Cross-cutting challenge**	**Number of responses reporting the challenge under each aspect of trial implementation**						
**Limited experience and capacity to undertake clinical trials**	5	4	1	1	2	1	2
**Underdeveloped infrastructure and/or inefficient processes**	4	1	12	9	5	1	3
**Limited communication/coordination and need for clear roles and responsibilities**	8	2		1			
**Cross-cutting lesson learned/best practice**	**Number of responses reporting the best practice/lesson learned under each aspect of trial implementation**						
**Sufficient training, capacity building, and supportive supervision**	4	5	5	1	3	1	1
**Sufficient infrastructure and/or streamlined processes**	2		3	3			2
**Good communication/coordination and clear roles and responsibilities**	6	1	2	3	4		1

^a^The denominator for the number of responses for each aspect of clinical trial implementation varies and is a subset of the 47 total responses. Participants were asked to select all aspects of trial implementation for which a guidebook on lessons learned/best practices they thought would be the most beneficial for trial sites. For each aspect selected, they were then asked to list up to three challenges and best practices/lessons learned but not every respondent listed challenges and/or best practices/lessons learned. Responses were categorized by theme, and the table captures the number of responses that fit within the three cross-cutting themes

^b^Respondent designated

### Training and regular supportive supervision to build capacity of site staff and other local stakeholders is important for the success of clinical trials

Gaps in knowledge and experience for site-level staff across multiple trial implementation aspects were identified as a challenge by 16 (34%) respondents. Inadequate training was identified as a challenge for trial administration. A lack of resources for local lab capacity building was reported as a challenge for laboratory strengthening/infrastructure. Limited experience and capacity of stakeholders was also reported as a challenge to implementation of community engagement at trial sites.

Lack of experience, lack of specific guideline[s], [and] difficulty measuring impact [made community engagement challenging]- Site Principal Investigator

With regard to ethics and regulatory requirements, limited experience of regulators and ethics committees with complex trials such as STREAM was identified as a contributing cause of long review periods and delays with regulatory approvals at some sites. And one respondent identified insufficient Sponsor site visits for supportive supervision as a challenge.

Ensuring there is local capacity building, adequate training, and ongoing supportive supervision during trials were identified as best practices that facilitate trial implementation by 20 (43%) respondents across several aspects of trial implementation. One site reported that their laboratory capacity improved through the use of a new method for TB diagnosis required by the STREAM protocol, and the laboratory planned to implement this methodology for programmatic use.

FDA [fluorescein diacetate] staining method [a new microbiology lab method of TB diagnosis] is a great achievement and [we] gained knowledge thanks to STREAM trial. Our lab plans to implement the methodology for programmatic use as well. [This was a] good example of capacity building. – Sub-investigator

One respondent reported that training pharmacy staff and regular Sponsor visits were an important part of successful trial implementation. Similarly, capacity building of community advisory board members was important for the trial, especially for sites where community engagement was new. Investing in training to improve and build capacity of local bodies and authorities around pertinent regulatory matters was also thought to be important for the success of research activities.

Patience, capacity building at [Regulatory Authorities] RA & [Ethics Committee(s)] EC is a must to continue trials. – Trial investigator

Knowledge gained through STREAM was applicable to programmatic activities and other trials.

[This was a] great experience for trial coordinators and administrators. The knowledge can be applied in daily [activities] and other trials as well. – Sub-investigator

### Investment in site-level infrastructure and well-designed trial processes is important for the success of clinical trials

Thirty-five (74%) respondents identified underdeveloped infrastructure and/or inefficient processes at trial sites as a significant challenge across all aspects of trial implementation. Infrastructure challenges were common for laboratory and pharmacy facilities; inefficient processes related principally to data management systems and processes. Respondents reported data management challenges including the use of paper data collection forms, double data entry, repeat data queries and long periods of time for resolution of data queries. Additionally, multiple respondents reported that intense paperwork including case report form documentation were difficult.

Use of paper CRF requiring double data entry made this study more labour intensive than necessary to capture the data. – Principal Investigator

Not having a dedicated onsite laboratory for the trial and needing to share a laboratory with the national lab system, lack of necessary calibration services, and lack of culture and LPA capacity were identified as challenges, as was the lack of an electronic lab register that was linked to the site. Long turnaround times for results were also reported as a challenge.

Reported pharmacy challenges included were related to local supply chain management including appropriate drug storage conditions and temperature monitoring at frontline health facilities. Lack of an electronic drug tracking system for the trial also made drug accountability difficult as noted by one respondent. Complex regulatory requirements for drug importation also made supply chain management complex.

Investment in infrastructure, including physical infrastructure and trial systems and processes, was reported as a best practice/lesson learned by 10 (21%) respondents across four aspects of trial implementation.

In terms of trial administration, frequent site monitoring visits and comprehensive knowledge of regulatory requirements, including import/export requirements, were identified as best practices.

Be familiar with import/export regulatory requirements and contracting out some of the processes such as obtaining import permits and customs clearance – Principal Investigator

Having adequate supplies of reagents and supplies, and meticulous checks and calibration procedures were identified as best practices/lessons learned for trial laboratories.

Investment in efficient supply chains, including cold chain infrastructure for appropriate storage and handling of drugs, to ensure an uninterrupted supply of trial medicines was identified as a best practice. The use of a pharmacy plan to clearly outline the process for medication dispensing, labeling, and monitoring was also identified as a best practice.

### Good communication and coordination between trial team members and regular communication with key stakeholders are vital to successful implementation

The third theme identified as a challenge across multiple aspects of trial implementation was the complexity of coordination and communication among stakeholders, as identified by 11 (23%) respondents.

At the beginning of the trial, it was challenging for all teams, such as the clinic team … to work with district TB dispensary MDR-TB staff because discharged patients were followed by the district TB staff. Once discharged, the clinic staff … have no connection or communication with the patients. – Trial Coordinator

Frequent communication and coordination among stakeholders and clearly defining roles and responsibilities for all stakeholders were identified as best practices across multiple aspects of trial implementation, including trial administration, pharmacy and supply chain management, and community engagement as noted by 17 (36%) respondents.

Coordination between the [National TB Program], indoor wards and DOTS providers [allowed] for seamless and uninterrupted provision of trial medicines. – Principal Investigator

Site staff (clinic, pharmacy, lab, data and district TB staff and DOT supervisors) work very closely to ensure quality care for the patients and their families. Team members learnt to delegate and perform their task and duties with high responsibilities. – Trial Coordinator

Regular and timely communication with regulatory authorities and ethics committees was also identified as a best practice.

EC members are happy with STREAM work at the [site]...during the site visits and in the meetings, the EC members appreciate timely reporting and regular update[s] from the site. – Trial Coordinator

### Recommendations for future trials

When asked to describe what they would do differently or how they would improve implementation of future trials at their sites, similar themes were identified. Fifteen (32%) respondents made recommendations related to training, capacity building, and ongoing supportive supervision and monitoring for site staff. One respondent reported a need for more time to review the protocol, individually, and as a team to address questions, and for ongoing discussions with the Sponsor before and after the site initiation visit. Similarly, another respondent said that questions related to site staff roles and responsibilities should be raised and answered at the site initiation visit. Training at the site initiation visit should not be limited to the protocol, but should cover all key topics relevant to efficient trial administration.

In addition to [the] protocol, CRFs and clinical management training at the site initiation, I think it is also important to give some guidance on reporting requirement[s], financial administration, specific requests due to the funding organization (USAID), etc. – Sub-investigator

Thirteen (28%) respondents recommended investments in infrastructure and trial processes, including institutions and systems, prior to trial implementation.

Establish the required financial and human resource management system before trial initiation. – Principal Investigator

Respondents identified a need for more efficient data management systems and collaboration with sites to ensure systems are adapted to the local context. Two respondents from one site, a data manager and a trial coordinator, reported that data entry systems and the database infrastructure should be updated for future trials. Drug accountability systems also need to be adapted to avoid duplicate data entry systems.

The third recommendation related to communication and coordination. Seven (15%) respondents said they would ensure early and increased communication with all stakeholders. For future trials, it would be important to improve communications with regulatory authorities and ethics committees, and coordination among trial staff was identified as essential for trial implementation.

Communicate with [the] Ministry of Health before deciding to participate to get better support. Submit the protocol for approval with [a] self-explanatory cover letter, [and] improve the relationship and communication with [the] RA & EC. – Investigator

In future trials, respondents also want an opportunity to participate in protocol development and provide more input on medical management of patients.

## Discussion

The findings from this study highlight trial site staff perspectives on challenges and best practices/lessons learned for MDR-TB clinical trial implementation drawn from the STREAM clinical trial experience. Survey responses from 87% of STREAM sites, covering settings in Europe, Africa, and Asia, provided a broad range of information and perspectives. Despite operating in settings with vastly different resources, capacities, and research experience, respondents from these diverse STREAM sites identified similar challenges and lessons learned/best practices related to investment and capacity building applicable across multiple aspects of trial implementation. This is consistent with previous studies, which concluded that sites in LMICs, where the burden of MDR-TB is highest, require additional investment and capacity building [3]. This indicates that Sponsors may need to alter traditional funding models to ensure sites have adequate funding to undertake required improvements before trial commencement.

Our results suggest that prior experience should be considered when planning training and capacity building activities, and additional time may be required for less experienced sites. When looking at the results based on whether a site had recent TB trial management experience, similar challenges were identified. However, when comparing sites with recent TB trial management experience to those without, more sites without recent experience reported that lack of CE experience was a challenge (three sites vs one site, respectively). Delays with approvals from regulatory authorities was also reported both by sites with and without recent TB trial management experience, and this may be reflective of research capacity and experience at the national level rather than the sites themselves. Our findings also highlight the importance of developing comprehensive training plans in collaboration with sites to ensure all site staff are prepared for trial implementation. Providing sufficient time for trial site staff to review key materials and ensuring adequate opportunity for questions and discussion during trainings and site initiation visits is important. In addition to training, prior to trial implementation, adjusting the number of monitoring visits and tailoring them to site needs can help ensure identified gaps are addressed. Investment in training, monitoring, and capacity building are critical for successful trial implementation.

Challenges related to underdeveloped infrastructure and inefficient trial processes also emerged across multiple trial implementation aspects. STREAM trial sites have vastly different experience conducting clinical trials and investment in infrastructure may need to be adapted for each site. Processes for different aspects of trial implementation, from drug accountability to collection and reporting of data, need to be clear and reviewed with sites prior to implementation to increase efficiency and ensure any limitations are identified and accounted for. Additionally, trial sites must be equipped with appropriate infrastructure prior to trial initiation, and our findings also demonstrate the importance of effective and regular communication and coordination among all stakeholders for successful implementation.

Our findings are comparable to previous reports on implementation of TB clinical trials [8, 14]. Schluger et al. outlined infrastructure needs including adequately experienced clinical and ancillary staff and functional laboratories to conduct TB trials in both low and high-burden countries [8]. These recommendations were also reflected as lessons learned from the field experience of a global clinical trial capacity-building program implemented in the context of trials for delamanid [14].

The results of our study were also similar to those from implementation of clinical trials for other diseases set in comparable settings [15–17]. Limitations in human resources and physical infrastructure were reported as critical setbacks in study implementation in Botswana, where sharing and coordination of existing resources between different research groups and partnerships were recommended as a strategy to overcome these challenges [15]. In a trial of treatment of cryptosporidiosis among adults with HIV infection in Malawi, identification of staff and infrastructure limitations related to laboratory systems were identified as challenges [16]. Overall, the themes that emerged in our analysis of challenges and lessons learned from trial implementation mapped well to those described in other studies, as well as to the unifying themes described in a systematic review that assessed barriers for conducting clinical trials in LMIC countries [5, 15–17]. It is also important to tailor efforts to the local context and type of trial. Stakeholder engagement is critical for addressing regulatory challenges, as is understanding and addressing infrastructure needs prior to trial initiation, and adapting study implementation to local considerations as required.

The current drug pipeline for TB is robust after nearly 40 years of inactivity, and regimens for drug-resistant TB are changing rapidly. Despite this, the body of clinical trial evidence to support new regimen recommendations remains limited, with many new recommendations made based on observational data. In order to generate high-quality evidence to guide regulators and policymakers to deliver effective and tolerable treatments for drug-resistant tuberculosis, it will be necessary to increase the number of sites able to conduct multi-site clinical trials; in turn, this will require investments in sites to carry out clinical trials [18].

In addition to building the capacity for and promoting research and trial conduct locally, these investments in high-burden settings often also feed into better management of the disease clinically as well as programmatically [15]. This was also seen in our results as respondents noted that the knowledge and skills gained from implementation of STREAM could be applied programmatically. Furthermore, the availability of locally generated evidence translates into better and quicker policymaking, as seen in the case of greater buy-in for research at policy levels [11].

There were limitations to our study. First, the survey instrument was not piloted, and its validity and reliability are therefore unknown. A majority of responses to open-ended survey questions were very brief, often limited to a few words, especially for best practices/lessons learned; thus, thematic analysis of these data was challenging. The use of structured interviews or focus group discussions would likely have resulted in richer qualitative data. The survey was conducted in English and although most trial staff are fluent in English, this may have led to selection bias. Limited English fluency may have also contributed to brief responses to the open-ended survey questions. Second, we analyzed the data from respondents individually, rather than looking at responses by site thus site-specific results may not be accurately captured. Third, the survey response rate is unknown. We do not have information on the total number of staff asked to complete the survey as the initial emails with the survey were only sent to a purposive sample of trial key personnel who were then asked to further disseminate to their teams. The two Stage 1 sites that did not participate in Stage 2 were already closed at the time the survey was distributed and it is possible that the survey was not shared with any additional staff at these two sites. The number of staff at each site varied, but the average estimated response rate is 12%, but this is likely an underestimate. Fourth, the large number of responses from three sites limits the generalizability of the findings to other sites, but all three reported challenges with supply chain management for trial drugs and challenges with trial coordination and coordination among stakeholders despite only one having TB trial management experience within 5 years of initiation of STREAM. Fifth, although respondents’ names were not collected, it is possible that results were impacted due to collection of trial site and role particularly when there was only staff member with a given role at a site (e.g., trial principal investigator). It is likely that the impact was minimal given that many roles at trial sites were redundant (e.g., sub-investigator, trial nurse). Despite the limitations, the study contributes important information from the perspective of diverse trial site staff from a variety of settings from their participation in a phase 3, registration trial of this scale.

## Conclusion

Site perspectives on clinical trial implementation are an important and often-neglected area of work that need to be documented and disseminated to sponsors, other sites, and implementation partners. Our research suggests that existing frameworks for clinical trial implementation should be reevaluated; successful implementation of trials may require increased upfront investments in capacity building, infrastructure, and trial processes; more collaboration with sites to improve systems and processes adapted to the local context; and upfront and ongoing stakeholder engagement.

### Supplementary Information

**Additional file 1. **Standards for Reporting Qualitative Research (SRQR)*.

## Data Availability

The datasets generated and/or analyzed during the current study are not publicly available due to collection of identifiable information of survey respondents but are available from the corresponding author on reasonable request.

## Appendix

Vital Strategies, the Sponsor of the STREAM clinical trial, is conducting a voluntary survey of principal investigators, trial coordinators, pharmacists, and other staff (e.g., laboratory staff, trial managers, etc.) at STREAM sites, to obtain and document information on commonly encountered challenges and lessons learned/best practices from implementation of the trial. Your participation in this survey is completely voluntary and whether or not you participate, it will have no impact on your site’s participation in the STREAM trial.

The information you provide will be kept confidential, and only researchers at Vital Strategies, the Medical Research Council Clinical Trials Unit at UCL, the Institute of Tropical Medicine, and the Liverpool School of Tropical Medicine will have access to the information. Results of the survey will be analyzed and disseminated in an aggregate form via reports and peer-reviewed publication. Although responses from a site may be used to demonstrate specific challenges or lessons learned, your individual information will not be shared in any form.

If you consent to participate in this voluntary survey, please indicate Yes below. If you do not consent, please select No below.

I. Demographics
  1. Please provide your role in STREAM
  2. Please select your STREAM site
II. Please select the topics below (note: there can be more than 1) for which a guidebook on lessons learned/best practices that you think would be the most beneficial for trial sites
  1. Laboratory strengthening/infrastructure
  2. Pharmacy and supply chain management
  3. Community engagement
  4. Regulatory requirements and ethics committee approvals
  5. Trial administration
  6. Health economics
  7. Other (please specify)
III. Challenges
  1. For each topic you selected above, please describe up to three challenges encountered with implementation of the STREAM trial (300 words)
IV. Best practices/lessons learned
  1. For each topic you selected above, please describe best practices/lessons learned from implementation of the STREAM trial
  2. If your site were to participate in another trial, please describe up to three things you would do differently/how you would improve clinical trial implementation at your site.

## Abbreviations

CRO: Contract Research Organization
STREAM: Evaluation of a Standardized Treatment Regimen of Anti-tuberculosis Drugs for Patients with Multidrug-resistant Tuberculosis
LMIC: Low- and middle-income countries
MDR-TB: Multidrug-resistant tuberculosis
TB: Tuberculosis
